# The Impact of Digital Mental Health Services on Loneliness and Mental Health: Results from a Prospective, Observational Study

**DOI:** 10.1007/s12529-023-10204-y

**Published:** 2023-07-24

**Authors:** Kirby Magid, Sara J. Sagui-Henson, Cynthia Castro Sweet, Brooke J. Smith, Camille E. Welcome Chamberlain, Sara M. Levens

**Affiliations:** 1 University of North Carolina at Charlotte, NC Charlotte, USA; 2 Modern Health, San Francisco, CA USA

**Keywords:** Loneliness, Mental Health, mHealth, Engagement

## Abstract

**Background:**

Loneliness has increased since the COVID-19 pandemic and negatively impacts mental health. This study examined relationships between loneliness and mental health among adults using a digital mental health platform.

**Methods:**

A purposive sample of 919 participants (97% response rate) who were newly enrolled in the platform completed a survey on loneliness, depression, anxiety, well-being, stress, social support, and comorbidities at baseline and 3 months. Platform engagement was tracked during this period. We examined baseline differences between lonely and non-lonely participants; associations between loneliness, mental health symptoms, and comorbidities; and changes in loneliness and mental health through engagement in any form of care.

**Results:**

At baseline, 57.8% of the sample were categorized as lonely. Loneliness was associated with younger age, fewer years of education, and the presence of a comorbidity (*p* values < .05). Baseline loneliness was associated with greater depression, anxiety, and stress and lower well-being and social support (*p*s < .001). The percentage of lonely participants decreased at follow-up (57.6% to 52.9%, *p* = .03). Those who improved in loneliness improved in mental health symptoms, well-being, and social support (*ps* < .001). Lonely participants who engaged in any form of care reported a greater reduction in loneliness than those who did not engage (*p* = .04).

**Conclusions:**

This study confirms previous findings of the high prevalence of loneliness among adults and risk factors for increased loneliness. Findings highlight the potential of digital platforms to reach lonely individuals and alleviate loneliness through remote mental health support.

**Supplementary Information:**

The online version contains supplementary material available at 10.1007/s12529-023-10204-y.

## Introduction

Forty percent of US adults experienced symptoms of anxiety or depression in 2021 [1]. These mental health symptoms can lead to a multitude of negative outcomes, including greater disability and economic burden [2, 3], reduced quality of life [4], and an increased risk of morbidity and mortality [5, 6]. Loneliness, defined as a subjective indicator of perceived isolation [5], is a major risk factor for depression, anxiety, and other mental health symptoms [6, 7]. It is also a rising public health issue that has become more prevalent since the onset of the COVID-19 pandemic, with three in five US adults reporting loneliness [8]. Past research has shown that lonely adults are nearly seven times more likely to meet clinical criteria for moderate to severe depression compared to non-lonely adults [9].

The relationship between loneliness and mental health is well-established. Loneliness is related to depression, anxiety, stress, and general mental health in studies of undergraduate students [10–12]. One nationally representative study conducted in Germany on adults aged 35 to 74 years old found that 10.5% of adults reported some degree of loneliness, and greater loneliness was related to depression, anxiety, suicidal ideation, and more frequent healthcare visits [13]. Another study examining the impact of loneliness on health outcomes in French adults during the first COVID-19 lockdown found that loneliness was related to greater anxiety, insomnia, and depressive symptoms. They also found that the relationship between loneliness and anxiety was moderated by employment and working arrangements, such that those who were unemployed and working remotely reported greater anxiety [14].

In addition to increased loneliness and mental health symptoms, the pandemic posed new challenges for delivering mental health care, with most services rapidly shifting to remote, technology-enabled service delivery [15]. Technology-enabled mental health support that offers equitable access to critical interventions has the potential to mitigate loneliness and its impact on mental health [16–19]. From a resource perspective, digital solutions are more scalable than in-person interventions and address many of the personal barriers to seeking support, such as time constraints, financial costs, proximity to mental health services, and availability [20, 21].

Research on technology-enabled mental health support interventions show that virtual one-on-one support, group support, and self-guided resources can increase feelings of connection, social support, shared experiences and help create and maintain social relationships, all of which can reduce loneliness and its adverse effects on mental health [22, 23]. Digital mental health services can also foster social skills, coping skills, and change maladaptive perceptions and thoughts, which can decrease loneliness [24, 25]. Additionally, digital mental health services in the form of videoconferencing with a therapist and validated web-based and mobile-based applications have been effective in reducing symptoms such as anxiety and depression in adults with medium to large effect sizes [26–35]. Specific to working populations, digital mental health services provided as an employee benefit have been found to improve a variety of mental health symptoms and well-being [36, 37].

Although many digital mental health solutions have been developed to treat mental health symptoms, only a few have targeted loneliness in conjunction with mental health symptoms. To date, most of the studies assessing the efficacy of digital mental health services and interventions for loneliness have been small pilot studies, feasibility studies, and have focused on specific populations, such as those with psychosis, older adults, and youth [17–19]. In sum, this research demonstrates that digital mental health solutions can mitigate heightened loneliness and other negative mental health symptoms in vulnerable populations. However, whether loneliness and mental health symptoms can be reduced in general adult populations engaging in digital mental health services sponsored by their employer is unknown. Thus, there is a need to determine if digital mental health services impact loneliness and its relationship with mental health in working adults.

This present study examined the prevalence of loneliness among working adults with mental health symptoms and changes in loneliness and mental health symptoms from voluntary engagement in an employer-sponsored digital mental health platform. The study aims were threefold: (1) explore baseline differences in demographics and care preferences among participants with varying degrees of loneliness; (2) examine baseline associations between loneliness, mental health symptoms, and medical comorbidities; and (3) evaluate longitudinal changes in loneliness and mental health symptoms and the impact of voluntary engagement in mental health services.

## Method

### Design and Participants

This investigation was a prospective, observational study of adults from multiple organizations and newly enrolled in an employer-sponsored digital mental health platform [Modern Health]. Eligible participants were: 18 years or older; had access to a smartphone, tablet, or computer; worked for companies who contracted with the mental health platform to provide services to their benefits-eligible employees; and had initiated care in the digital care platform through matching with a therapist or coach and/or engaging with digital content. Data were collected between September 2021 and May 2022. The study protocol was reviewed and approved by Western Clinical Group Institutional Review Board.

### Procedure

Participants registered for a platform account through a connected device (i.e., through the platform mobile website or on the platform app). During onboarding, participants were able to select their preferences for care (e.g., 1:1 care, self-guided digital content, or small groups) and topics of focus (e.g., my relationships, my emotions, my professional life), as well as complete a number of validated clinical assessments to determine the severity of their current mental health symptoms. Based on this data, a proprietary algorithm recommended participants to an initial care plan.

Using purposive sampling, eligible individuals were invited via email to complete an initial research study screening questionnaire approximately 2 weeks after onboarding. The purpose of the screening questionnaire was to obtain additional demographic information to ensure a balanced sample at baseline across age groups, gender identities, race identities, and clinical acuities. After completion of the screening questionnaire, eligible individuals were provided additional information about the study and asked to provide informed consent to participate. Consenting participants were emailed a secure link to complete a baseline survey. Three months post-baseline, participants were asked to complete a follow-up survey. All surveys were hosted on a secure, online survey platform (Qualtrics, Provo, UT). Upon completion of each survey, participants were compensated with a $25 digital gift card.

### Digital Mental Health Services

The digital mental health platform [Modern Health] is a mental health benefit offered to eligible employees at no cost, with services paid for by the employer. The platform includes a variety of options for members to access care, including access to providers (licensed clinicians or professional coaches) for one-on-one sessions (recommended to members with a preference to receive care “one-on-one”), group psychoeducational sessions (recommended to members with a preference to receive care “with a small group”), and a comprehensive suite of evidence-based digital, self-guided resources and materials (recommended to members with a preference to receive care “on my own”). All one-on-one care was delivered remotely using a web browser or mobile device by (1) licensed mental health clinicians with advanced degrees/graduate training and licensure in mental health care and/or (2) International Coaching Federation accredited professional coaches. Therapists used a variety of widely accepted, evidence-based therapeutic approaches such as cognitive behavioral therapy, acceptance and commitment therapy, mindfulness-based stress reduction, and dialectical behavior therapy. All providers were vetted for their use of these evidence-based practices, and the number of sessions a participant attended was dependent on the allotted number of sessions covered by their employer and their therapeutic need. Participants also had access to unlimited online, group psychoeducational sessions facilitated by therapists or coaches and designed to address a range of topics, such as the intersection of mental health and identity, coping with stressful events or relationship issues. All participants had unlimited access to an on-demand, self-guided digital library of mental health programs and resources. This library included daily exercises, interactive programs and podcasts, mindfulness exercises such as meditations and breathing, and self-paced structured educational lessons. Digital resources were designed by an in-house team of clinical psychologists and covered topics including emotions, relationships, and professional life. Many resources targeted social health, such as strengthening relationships, building a strong and safe support system, finding community and belonging, navigating conflict with confidence, and social identity in relationships.

### Measures

#### Demographics

Participants provided information on their age, education level, gender identity, and race/ethnicity during the screening process. Participants were able to select all that applied from a list of 11 gender identity and 7 race/ethnicity options. Due to extreme skewness in the distribution, gender identity was dichotomized into man or woman. Forty-three participants identified as non-binary and were included in the baseline analyses. Race/ethnicity options were also dichotomized into BIPOC (Black, Indigenous, and People of Color) and White due to low numbers of participants in the non-white race categories and for ease of analysis.

#### Care Modality Preference

Preference for mode of receiving care was collected during the onboarding process. Participants were asked to report their preferred format in which to receive mental health care from the following response options: “On my own,” “With a small group,” “One-on-one,” or “I’m not sure.”

#### Primary Topic of Focus

Participants were asked to choose from over forty topics of focus or reasons for seeking care during the onboarding process. These topics were wide-ranging and categorized along the following dimensions: “my emotions,” “my professional life,” “my physical well-being,” “my relationships,” and “my finances.” After choosing their topics, individuals were prompted to choose one topic to focus on first.

#### Loneliness

Loneliness was measured using the 3-item UCLA Loneliness Scale [38] at baseline and 3-month follow-up. Participants responded on a 3-point scale (1 = Hardly ever; 3 = Often) to items designed to assess loneliness. Item responses were summed to create a total continuous score ranging from 3 to 9. A binary variable was also created to categorize participants as either lonely (denoting a total score > 5) or non-lonely (denoting a total score ≤ 5) based on previous research [39, 40]. Improvement in loneliness was indicated when a participant was classified as lonely at baseline (score > 5) but no longer classified as lonely at follow-up (score ≤ 5).

#### Depression Symptoms

The presence and severity of depression symptoms at baseline and 3-month follow-up was assessed with the 9-item Patient Health Questionnaire (PHQ-9) [41]. Participants responded on a 4-point scale (0 = not at all; 3 = nearly every day), and responses were summed to create a total continuous score ranging from 0 to 27, wherein higher scores reflect greater severity of depressive symptoms. For interpretation, a score of 0–4 was considered none to minimal, 5–9 was considered mild, 10–14 was considered moderate, 15–19 was considered moderately severe, and 20–27 was considered severe, as recommended by prior literature [41].

#### Anxiety Symptoms

The presence and severity of anxiety symptoms at baseline and 3-month follow-up was assessed with the 7-item Generalized Anxiety Disorder Questionnaire (GAD-7) [42]. Participants responded on a 4-point scale (0 = not at all; 3 = nearly every day), and responses were summed to create a total continuous score ranging from 0 to 21; a score of 0–4 was considered none to minimal severity, a score of 5–9 was considered mild, a score of 10–14 was considered moderate, and a score of 15–21 was considered severe symptomatology [42].

#### Well-Being

Well-being was assessed during onboarding and at 3-month follow-up with the 5-item World Health Organization Well-Being index (WHO-5) [43]. Participants responded on a 6-point scale (0 = at no time; 5 = all of the time), and scores were summed and multiplied by 4 to obtain a total continuous score ranging from 0 to 100. Higher scores indicate higher subjective well-being, with a score below 28 indicating high risk for depressive symptoms [26].

#### Stress

The 4-item Perceived Stress Scale (PSS-4) [44] was used to measure stress levels at baseline and 3-month follow-up. Participants responded on a 5-point scale (0 = never; 4 = very often). Item responses were summed to create a total continuous score ranging from 0 to 16. Higher scores indicate higher perceived stress.

#### Social Support

The Medical Outcomes Study Social Support Survey (MOS-SSS-6) [45] was used to measure participants’ perceived level of global functional social support. Participants responded on a 5-point scale (1 = none of the time; 5 = all of the time), and item responses were summed to create a total continuous score ranging from 6 to 30. Higher scores indicate more perceived social support.

#### Comorbidities

Participants were asked to provide information about their diagnostic history of major physical and mental health conditions. Participants were able to select all that applied from a list of 22 common physical and mental health conditions, such as attention-deficit/hyperactivity disorder, eating disorders, obesity, and diabetes, and/or report conditions not captured by our provided list through a free-text option. A binary variable was created to denote whether participants reported any pre-existing medical comorbidity (coded as 1) or no comorbidities (coded as 0).

#### Platform Engagement

Engagement metrics were tracked directly on the mental health platform. Engagement was defined as attending at least one session with a therapist or coach and/or at least one interaction with digital content (defined as accessing meditations, breathing exercises, self-paced structured psychoeducational reading lessons, interactive reading programs, and podcasts) throughout the 3-month follow-up period. Using this definition, a binary variable was created to categorize participants as either engaged (coded as 1) or not engaged (coded as 0).

### Data Analysis

Data were analyzed using R version 4.2.2 [46]. Descriptive statistics were generated to describe participants’ demographic characteristics and care preferences. To assess the first aim, independent samples *t*-tests and Pearson’s chi-squared tests were used to assess baseline differences in demographics and care preferences between lonely and non-lonely participants. To assess the second aim, bivariate correlations were used to examine baseline associations between loneliness and mental health symptoms (e.g., depression, anxiety, well-being, stress, and social support). We also conducted a logistic regression to estimate the relationship between having a medical comorbidity and loneliness at baseline.

To examine the third aim, McNemar’s chi-squared test for dependent samples was used to determine changes in the percentage of participants who were categorized as lonely at baseline compared to follow-up. A paired *t*-test was used to investigate changes in loneliness from baseline to follow-up among participants who were categorized as lonely at baseline. Additionally, paired *t*-tests were used to examine changes in mental health symptoms from baseline to follow-up among those who improved in loneliness at follow-up. To calculate percent improvement in loneliness and mental health symptoms from baseline to follow-up, mean follow-up scores were subtracted from baseline scores, and this value was then divided by the baseline score and multiplied by 100. A two-way repeated measures ANOVA was conducted to test whether longitudinal changes in loneliness differed between participants who were categorized as lonely at baseline and engaged in care and participants who were lonely at baseline but did not engage in care.

## Results

### Study Participants

Out of the 8786 individuals who were eligible and invited to participate in the study, 950 agreed to participate and provided informed consent. Of this sample, 31 had incomplete UCLA loneliness data, leaving a baseline sample of 919 adults (97%) with complete UCLA loneliness data. Out of the 703 adults who completed the follow-up survey, 13 were excluded due to incomplete UCLA loneliness data, leaving a final follow-up sample of 690 (98%) available for analysis. Analysis of baseline data on individuals who had complete data in the follow-up survey (*n* = 690; “completers”) versus those who did not (*n* = 229; “non-completers”) revealed no significant differences in age, gender identity, race/ethnicity, or loneliness at baseline. Completers had significantly higher baseline depression (*p* = 0.004), anxiety (*p* = 0.02), and stress (*p* = 0.03), and lower well-being (*p* = 0.01) than non-completers.

To address the study aims, baseline analyses were conducted using the sample of 919 adults and longitudinal analyses were conducted using the sample of 690 adults. Descriptive statistics and demographics for the baseline sample are reported in Table 1. Demographics for the longitudinal sample are reported in Table S1 of the Electronic Supplementary Materials.

**Table 1:** Baseline demographic characteristics between lonely and non-lonely participants

**Variable**	**Total** ***n*= 919**	**Lonely** ***n*= 531**	**Non-Lonely** ***n*= 388**	***t***	***χ2***	***df***	***p***
Age, *M (SD)*	33.88 (8.78)	33.35 (8.78)	34.62 (8.74)	2.16		917	.031
Gender, *n* (%)					4.56	2	.102
Woman	544 (59.2)	316 (59.5)	228 (58.8)				
Man	330 (35.9)	182 (34.3)	148 (38.1)				
Non-binary	43 (4.7)	31 (5.8)	12 (3.1)				
Declined to answer	2 (0.2)	2 (0.4)	–				
Race/ethnicity, *n* (%)					.89	1	.346
BIPOC	381 (41.5)	213 (40.1)	168 (43.3)				
White	535 (58.2)	317 (59.7)	218 (56.2)				
Declined to answer	3 (0.3)	1 (0.2)	2 (0.5)				
Education, *n* (%)					9.31	2	.009
< Bachelors	135 (14.7)	88 (16.6)	47 (12.1)				
Bachelors	530 (57.7)	315 (59.3)	215 (55.4)				
> Bachelors	254 (27.6)	128 (24.1)	126 (32.5)				
Topic of focus, *n* (%)					19.19	4	< .001
Emotions	454 (49.4)	285 (53.7)	169 (43.6)				
Professional life	135 (14.7)	60 (11.3)	75 (19.3)				
Physical well-being	92 (10.0)	46 (8.7)	46 (11.9)				
Relationships	205 (22.3)	125 (23.5)	80 (20.6)				
Finances	32 (3.5)	15 (2.8)	17 (4.4)				
Missing/not available	1 (0.1)	–	1 (0.3)				
Care preference, *n* (%)					6.58	3	.086
On my own	96 (10.4)	45 (8.5)	51 (13.1)				
1:1	550 (59.8)	335 (63.1)	225 (58.0)				
Small group	26 (2.8)	13 (2.4)	13 (3.4)				
Not sure	216 (23.5)	129 (24.3)	87 (22.4)				
Missing/not available	21 (2.3)	9 (1.7)	12 (3.1)				

*BIPOC* Black, Indigenous, and People of Color. Lonely =  > 5 on the UCLA loneliness scale; non-lonely ≤ 5

### Baseline Differences in Demographics and Care Preferences

At baseline, 57.8% (*n* = 531) of the sample were categorized as lonely. Lonely participants were slightly younger, *t*(917) = 2.17,* p* = 0.03, and had fewer years of education than non-lonely participants, *χ2* = 9.31, df = 2, *p* = 0.009. There were no significant gender, race/ethnicity, or care preference differences (Table 1). Lonely participants were more likely to select “my emotions” as their topic of focus (*p* = 0.02); non-lonely participants were more likely to select “my professional life” (*p* = 0.006).

### Baseline Associations Between Loneliness, Mental Health, and Comorbidities

At baseline, greater loneliness was associated with greater symptoms of depression (*r* = 0.49, *p* < 0.001), anxiety (*r* = 0.39, *p* < 0.001), and stress (*r* = 0.42, *p* < 0.001), and lower well-being (*r* =  − 0.38, *p* < 0.001) and social support (*r* =  − 0.59, *p* < 0.001). People with any medical comorbidity were at greater risk of being lonely (*β* = 0.67, OR = 1.95, 95% CI 1.41, 2.71, *p* < 0.001, *n* = 912). Additionally, when splitting the analysis by mental and physical comorbidity, both significantly predicted higher odds of being lonely (see Table S4 in Electronic Supplementary Materials). Because associations between age, gender, race/ethnicity, education, and loneliness were weak to non-significant, we did not include them as covariates in these analyses.

### Longitudinal Changes in Loneliness, Mental Health, and Platform Engagement

In the longitudinal sample, McNemar’s test revealed a significant decrease in the percentage of lonely participants from baseline (*n* = 396; 57.4%) to follow-up (*n* = 368; 53.3%), *χ2* = 4.78, *p* = 0.03. Those who were categorized as lonely at baseline reported a significant decrease in loneliness from baseline (*M* = 7.09, *SD* = 1.14) to follow-up (*M* = 6.37, *SD* = 1.57), *t*(395) = 10.25, *p* < 0.001, representing an 10% improvement, though still remaining in the lonely range (scores > 5). Those who were categorized as improving in loneliness at follow-up (*n* = 96, 13.9%) reported significant reductions in depression, anxiety, and stress and significant increases in well-being and social support (*p*s < 0.001; Table 2). Specifically, participants reported a 38% reduction in depressive symptoms, a 37% reduction in anxiety symptoms, a 20% reduction in stress, 28% increase in well-being, and an 8.5% increase in reported social support. Because associations between demographics and mental health symptoms were weak (< 0.3) to non-significant at follow-up, we did not include them as covariates in our analysis.

**Table 2:** Changes in mental health symptoms among those who improved in loneliness

**Variable**	**Range**	**Baseline** ***M*(***SD***)**	**Follow-up** ***M*(***SD***)**	***t***	***p***
Depression (PHQ-9)	0–27	8.33 (5.04)	5.06 (4.11)	7.35	< .001
Anxiety (GAD-7)	0–21	7.97 (5.23)	5.00 (4.11)	6.47	< .001
Stress (PSS-4)	0–16	7.18 (2.45)	5.72 (2.43)	5.51	< .001
Well-being (WHO-5)	0–100	45.58 (17.98)	58.21 (19.84)	− 6.43	< .001
Social Support (MOS-SSS-6)	6–30	23.51 (5.05)	25.52 (4.92)	− 4.33	< .001

*n* = 96, df = 95. Tests were performed on the subsample of participants who improved in loneliness from baseline to follow-up. Improvement in loneliness was indicated when participants met or exceeded the UCLA Loneliness scale cutoff (> 5) at baseline and were below the cutoff at follow-up (≤ 5). Higher scores = higher levels of each construct

A two-way repeated measures ANOVA was conducted to test if engaging with the digital mental health platform was associated with a significant decrease in loneliness over time among people who were categorized as lonely at baseline (*n* = 396). Of the participants who were lonely at baseline and had available 3-month data, 306 (77%) participants engaged with the platform, and 90 (22%) participants did not engage. There were no significant differences in age, race/ethnicity, education, or gender between lonely participants who engaged with the platform and those who did not. There were also no significant associations between loneliness at follow-up and demographics; therefore, we did not control for demographics in longitudinal analyses. Of those who engaged, 106 (35%) attended at least 1 therapy session, 169 (55%) attended at least 1 coaching session, 204 (67%) used at least 1 piece of digital content, and 44 (14%) registered for a group session. There was a statistically significant difference in loneliness at follow-up between those who engaged (*M* = 6.33, *SD* = 1.58) and those who did not engage (*M* = 6.52, *SD* = 1.57), (*F*(1,394) = 4.14, *p* = 0.04, Table 3). Participants who engaged in care demonstrated a greater reduction in loneliness (− 0.79 points) at follow-up compared to those who did not engage (− 0.46 points).

**Table 3:** Two-way repeated measures ANOVA of change in loneliness by engagement

**Factor**	**Sum of squares**	**df**	***F***	***p***
Engaged	.1	1	.03	.854
Time	101.8	1	105.82	< .001
Engaged x Time	4	1	4.14	.042

*n* = 396. Test was performed in participants who met the cut-off for higher loneliness at baseline (> 5 on the UCLA Loneliness scale). Time = from baseline to follow-up

## Discussion

This study examined the prevalence of loneliness and mental health symptoms and longitudinal changes in these outcomes among people seeking support from an employer-sponsored digital mental health platform. More than half of the sample at baseline reported high levels of loneliness, and greater loneliness was associated with poorer mental, physical, and social health before initiating care. Over a 3-month follow-up period, rates of loneliness significantly decreased, but there remained room for improvement. Participants categorized as lonely at baseline who engaged with digital mental health services over time had the greatest reductions in loneliness, and those who were no longer categorized as lonely at follow-up reported significant improvements in mental and social health. These findings re-emphasize the latent presence of loneliness, particularly among individuals seeking mental health support and living with concomitant mental health symptoms of anxiety and depression. Loneliness is multi-faceted, as evidenced here by its connections with biopsychosocial health, and our results indicate that connecting with digital mental health services can play an important role in detecting and alleviating loneliness and improving other aspects of mental health and well-being.

Our first aim was to explore the baseline prevalence of loneliness and any differences in demographics and care preferences among participants with varying degrees of loneliness. Almost three in five people (58%) seeking care from the digital mental health platform reported feeling lonely. It is possible this could be due to COVID-19 social distancing strategies and increases in remote work during the time in which this study was conducted [47, 48]. However, American adults were already facing mental health challenges before the COVID-19 pandemic, with national rates of loneliness between 47 and 61% [8]. Recent studies have shown these rates have either risen [49], for example, among young adults, people with lower incomes, and those from minoritized groups, or have remained steady, especially among those with comorbid mental health concerns [50]. Our findings mirror these national statistics where participants classified as lonely before starting care were more likely to be younger and have fewer years of formal education compared to those not classified as lonely, though the strength of these correlations was small. Young adulthood is a particularly vulnerable time to experience social distancing because this is a critical period of interpersonal development and the formation of a support network outside one’s family or school group [51].

Our second aim was to examine how loneliness was related to other mental, physical, and social health factors. Greater loneliness was associated with higher depression, anxiety, and stress and lower well-being, supporting prior research connecting loneliness to mental health challenges [6, 52]. Chronic loneliness can increase negative self-beliefs, lead to or exacerbate social anxiety, and reduce life satisfaction [53]. It can also heighten biological stress and inflammatory processes during stressful situations [54]. With regard to social health, baseline loneliness was related to lower functional social support, meaning people with greater feelings of loneliness may have perceived they had fewer people to help them with things like illness, personal problems, and sharing feelings and enjoyable experiences [55]. The lack of quality social connections is different from, but related to, perceived loneliness, and the link between the two can vary: the lack of social connections can lead to loneliness in some people, while others can feel lonely despite having a social network [55, 56]. There was an association with a large effect size in this study highlighting the possibility that social support may be a protective factor in treatment seeking populations.

Participants with any diagnosed medical comorbidity were also almost two times more likely to be classified as lonely compared to those without a comorbidity. In addition to being linked to mental health concerns like anxiety and depression, previous research has connected loneliness to all-cause mortality potentially through physical health mediators [7]. The directionality of this link remains unclear with some research finding that being less socially connected leads to poorer health and lower survival rates [57], yet having a preexisting health condition may lead to feelings of loneliness because one’s health limits their ability to socially connect [58]. The findings from this study are consistent with prior work around the effects of loneliness on health and emphasize the urgency in reducing loneliness stigma, routinely assessing loneliness during clinical encounters, and developing solutions to help people feel more connected.

Our third aim was to evaluate longitudinal changes in loneliness and mental health symptoms and the associated impact of engaging with digital mental health services. The proportion of participants with loneliness significantly decreased after 3 months, yet the prevalence remained high at almost 53%. Those who were classified as lonely at baseline reported a significant 10% improvement by 3 months. This suggests that engagement in digital mental health care can improve loneliness to some extent, but the magnitude of improvement is modest. Given the short time frame (3 months) and the fact that the services did not specifically target loneliness, it is understandable that the magnitude of the change in loneliness was modest but detectable. Alleviating loneliness does not happen solely through the efforts of an individual; interventions need a socioecological approach to also target community and work environments and sufficient time for social connectedness to solidify [59].

Participants classified as lonely at baseline who engaged with any digital mental health services had the greatest reductions in loneliness. When the COVID-19 pandemic began, not only did social distancing drastically increase but most mental health care shifted to virtual formats, still leaving over half of the population with any mental health concern without treatment in 2021 [60]. The digital mental health platform studied here is unique because it provided several different types of remote services within a single platform and accelerated access to vital resources during a time of social isolation. Using Levesque et al.’s framework of healthcare access [61], the services studied here promoted increased access by being approachable (as an employee benefit), acceptable (the care was evidence-based and culturally centered), available (providers were available for sessions within ~ 1 day and there were on-demand resources), affordable (as an employer-sponsored, free benefit), and appropriate (with high engagement rates and clinical effectiveness, e.g., [62]).

Engaging with accessible digital mental health services can facilitate opportunities to connect with others through group sessions and one-on-one support, which may help curb loneliness [63, 64]. Many of the self-guided digital resources are psychoeducational and covered topics targeting social health, such as strengthening relationships and building a strong and safe support system. The group psychoeducational sessions may have also fostered a sense of support and social connectedness as participants had tangible access to others sharing similar experiences. Working one-on-one with a mental health provider may have helped participants build and reinforce social connectivity at work and in their community, increased their positive feelings about their personal relationships, or help them balance daily activities (e.g., sleeping or working too much or too little) that impede one’s mental health and their ability to have meaningful social interactions [65, 66].

Those whose loneliness improved and were no longer categorized as lonely at follow-up also reported significant improvements in mental and social health, with the greatest amelioration in depression and anxiety symptoms (39% and 37% improvement, respectively) and substantive improvements in stress and well-being (20% and 28%, respectively). This link between loneliness and improved mental health outcomes is consistent with prior research, including the longest running scientific study of happiness, health, and aging (the Harvard Study of Adult Development) which found that the strongest predictor of positive mental health and well-being over time was the quality of a person’s social connections [67].

The smallest improvement was in reported functional social support (8.5% increase). As the focus of the digital mental health services studied here were on the individual, perceptions or coping with loneliness may improve but not necessarily change ecological factors, or availability and quality of social connections. Further, changes in loneliness and social isolation are often only moderately correlated [68]; people who are socially isolated do not universally experience loneliness and people who have large social groups can still report feeling lonely. Overall, our results highlight the multifaceted nature of loneliness and the complexity of relationships between loneliness, overall mental health and well-being, and other social factors.

### Limitations

This study had several limitations. First, the study was observational and although we used real-world data, we were not able to make causal claims. While we can infer an association between engagement in digital mental health services and loneliness and mental health symptoms, without random assignment and control of exposure, we cannot determine a definite cause of the improvement, or if one modality was more or less impactful than another (e.g., does working with a provider improve loneliness more than using self-guided digital content). Future research with an experimental design and comparison condition is needed to test these direct effects and to isolate the relative effectiveness of specific care modalities. Second, although improvements in loneliness were observed from baseline to follow-up and the accessible mental health services focused on transdiagnostic skills and emotional health, the platform services were not specifically designed to target loneliness or social connectedness; though these topics may have surfaced during the course of accessing care, we do not have data to explicitly identify if they did and how they were addressed. Third, there was a study loss-to-follow-up rate of 25% (919 at baseline to 690 at follow-up). Those with higher baseline symptoms of depression and anxiety were more likely to drop from the study, though it is common for people with higher mental health concerns to drop out in longitudinal studies [69]. Thus, we have diminished ability to examine longitudinal changes for those with more severe symptomatology. Fourth, engagement in digital mental health services was based on attending at least 1 session or having at least 1 interaction with digital content. More engineering around metrics of engagement (such as cumulative time spent on the platform, or frequency of interaction) would enable us to examine more refined associations and interactions. Fifth, the intervention was an employer-sponsored mental health benefit and only included adults employed by companies that offered the benefit. The findings may not be generalizable to other employee populations or contexts for working adults. It is possible that some employees with access were not comfortable engaging in mental health services offered through their employer due to privacy or confidentiality concerns, despite regulations in place to protect employees’ health information from the employer, and security/privacy safeguards built into the platform to protect identities and data. As a result, the employees in the analyzable sample may not be fully representative of all employees with access to the platform and services.

## Conclusion

Overall, this study highlights the persistent co-occurrence of loneliness with sub-optimal mental health. Given the known and insidious impact of loneliness on mental health, and the compounded impact of loneliness and mental health impairments on physical health and worker productivity [8], it is imperative to find acceptable and scalable solutions for detecting and alleviating loneliness and strengthening mental well-being.

Our results also offer encouragement that digital platforms are a viable opportunity. Digital solutions are scalable, can mold to fit the daily patterns of individuals (i.e., through their mobile devices), and provide options for individuals to engage in care privately and according to their preferences. Digital platforms also provide connection points for employees when in-person interactions are not feasible (e.g., during COVID), as well as when company workplace structure relies on remote work [48]. The present findings in conjunction with current changes in workplace structure and environment also provide a path for increasing belongingness at the workplace [70]. Employer sponsored digital healthcare opportunities may help participating employees to feel cared for by the company they work for, thereby increasing belongingness in the workplace, which may also counter feelings of loneliness. With the proliferation of digital platforms designed to address mental health and the increase in employers providing digital mental health options to their workforce [71], these solutions have great potential to reach large audiences and reduce structural and interpersonal barriers to addressing loneliness.

### Supplementary Information

Below is the link to the electronic supplementary material.

Supplementary file1 (DOCX 30 KB)

## Data Availability

Individual de-identified data that underlie the results reported in this manuscript can be shared privately for research purposes upon receipt of a methodologically sound proposal, and whose proposed use of the data from the study related to this article are approved by the authors. To gain access, requesters will need to submit a proposal to the corresponding author and sign a data access agreement that includes a commitment: (1) to using the data only for research purposes; (2) to not attempt to, or actually, re-identify any individual; (3) to securing the data using appropriate safeguards; and (4) to destroying or returning the data after analyses are completed.
