# Supplementary material for: The Impact of Digital Mental Health Services on Loneliness and Mental Health: Results from a Prospective, Observational Study
Source: Int J Behav Med. 2023 Jul 24;31(3):468–78. doi: 10.1007/s12529-023-10204-y (PMC11106110; doi:10.1007/s12529-023-10204-y)
Supplement: Supplementary file 1 — Supplementary file1 (DOCX 30 KB) [file 12529_2023_10204_MOESM1_ESM.docx]

Electronic Supplementary Materials

The Impact of Digital Mental Health Services on Loneliness and Mental Health: Results from a Prospective, Observational Study

This document includes the following supplemental materials: Page

1. Table S1. Demographic information for longitudinal sample.…………………………...2
2. Tables S2. Baseline presence of mental comorbidities…………………...……………....4
3. Table S3. Baseline presence of physical comorbidities…………………...……………...7
4. Table S4. Logistic regression of mental and physical health comorbidities predicting likelihood of loneliness……………………………………… …………...……………..10

**Table S1.**

*Demographic Characteristics of Longitudinal Sample (n = 690)*

| **Variable** |  |
| --- | --- |
| Age, *M (SD)* | 33.89 (8.86) |
| Gender, *n (%)* |  |
| Woman | 422 (61.2) |
| Man | 236 (34.2) |
| Non-binary | 32 (4.6) |
| Race/Ethnicity, *n (%)* |  |
| BIPOC | 281 (40.7) |
| White | 409 (59.3) |
| Education, *n (%)* |  |
| <Bachelors | 97 (14.1) |
| Bachelors | 408 (59.1) |
| >Bachelors | 185 (26.8) |
| Topic of focus, *n (%)* |  |
| Emotions | 344 (49.9) |
| Professional life | 97 (14.1) |
| Physical well-being | 75 (10.9) |
| Relationships | 148 (21.5) |
| Finances | 25 (3.6) |
| Missing/not available | 1 (0.1) |
| Care preference, *n (%)* |  |
| On my own | 74 (11.0) |
| 1:1 | 420 (62.2) |
| Small group | 19 (2.8) |
| Not sure | 162 (24.0) |
| Missing/not available | 15 (2.3) |
| Loneliness, *M (SD)* | 5.81 (1.80) |

| **Table S2.**  *Baseline Presence of Mental Comorbidities (n = 919)* | |
| --- | --- |
| **Comorbidity** | ***n (%)*** |
| Attention-Deficit / Hyperactivity Disorder (ADHD) |  |
| Absent | 806 (87.8) |
| Present | 112 (12.2) |
| Declined to answer | 1 (0.1) |
| Anxiety |  |
| Absent | 562 (61.2) |
| Present | 357 (38.8) |
| Autism |  |
| Absent | 913 (99.5) |
| Present | 5 (0.5) |
| Declined to answer | 1 (0.1) |
| Bipolar disorder |  |
| Absent | 883 (96.2) |
| Present | 35 (3.8) |
| Declined to answer | 1 (0.1) |
| Depression |  |
| Absent | 600 (65.3) |
| Present | 319 (34.7) |
| Eating disorder |  |
| Absent | 867 (94.4) |
| Present | 51 (5.6) |
| Declined to answer | 1 (0.1) |
| Personality disorder |  |
| Absent | 911 (99.3) |
| Present | 6 (0.7) |
| Declined to answer | 2 (0.2) |
| Postpartum mood disorder |  |
| Absent | 900 (98) |
| Present | 18 (2.0) |
| Declined to answer | 1 (0.1) |
| Obsessive Compulsive Disorder (OCD) |  |
| Absent | 881 (96.0) |
| Present | 37 (4.0) |
| Declined to answer | 1 (0.1) |
| Schizophrenia |  |
| Absent | 916 (99.8) |
| Present | 2 (0.2) |
| Declined to answer | 1 (0.1) |
| Substance use |  |
| Absent | 878 (95.6) |
| Present | 40 (4.4) |
| Declined to answer | 1 (0.1) |
| Posttraumatic Stress Disorder (PTSD) |  |
| Absent | 838 (91.3) |
| Present | 80 (8.7) |
| Declined to answer | 1 (0.1) |
| Mental comorbidities total |  |
| Presence of 0 | 448 (48.9) |
| Presence of 1 | 150 (16.4) |
| Presence of 2 | 159 (17.3) |
| Presence of 3 | 92 (10) |
| Presence of 4 | 44 (4.8) |
| Presence of 5 | 14 (1.5) |
| Presence of 6+ | 10 (1.0) |
| Insufficient information | 2 (0.2) |
| ***Note.*** Members missing data on any mental comorbidities were automatically grouped into the insufficient information group for mental comorbidities total. | |

| **Table S3.**  *Baseline Presence of Physical Comorbidities (n = 919)* | |
| --- | --- |
| **Comorbidity** | ***n (%)*** |
| Cardiovascular condition |  |
| Absent | 892 (97.2) |
| Present | 26 (2.8) |
| Declined to answer | 1 (0.1) |
| Cerebrovascular disease (including stroke, TIA) |  |
| Absent | 914 (99.6) |
| Present | 4 (0.4) |
| Declined to answer | 1 (0.1) |
| High blood pressure |  |
| Absent | 802 (87.4) |
| Present | 116 (12.6) |
| Declined to answer | 1 (0.1) |
| Obesity |  |
| Absent | 771 (84.0) |
| Present | 147 (16.0) |
| Declined to answer | 1 (0.1) |
| Sleep apnea |  |
| Absent | 856 (93.3) |
| Present | 61 (6.7) |
| Declined to answer | 2 (0.2) |
| Respiratory condition (including COPD, asthma) |  |
| Absent | 788 (85.9) |
| Present | 129 (14.1) |
| Declined to answer | 2 (0.2) |
| Diabetes |  |
| Absent | 884 (96.3) |
| Present | 34 (3.7) |
| Declined to answer | 1 (0.1) |
| Cancer |  |
| Absent | 893 (97.3) |
| Present | 25 (2.7) |
| Declined to answer | 1 (0.1) |
| Bone or joint condition |  |
| Absent | 849 (92.5) |
| Present | 69 (7.5) |
| Declined to answer | 1 (0.1) |
| Neurological condition |  |
| Absent | 905 (98.6) |
| Present | 13 (1.4) |
| Declined to answer | 1 (0.1) |
| Other condition |  |
| Absent | 753 (83.2) |
| Present | 152 (16.8) |
| Insufficient information | 14 (1.5) |
| Physical comorbidity total |  |
| Presence of 0 | 469 (51.9) |
| Presence of 1 | 237 (26.2) |
| Presence of 2 | 113 (12.5) |
| Presence of 3 | 51 (5.6) |
| Presence of 4 | 25 (2.8) |
| Presence of 5 | 7 (0.8) |
| Presence of 6+ | 2 (0.2) |
| Insufficient information | 15 (1.6) |
| ***Note****.* Members missing data on any physical comorbidities were automatically grouped into the insufficient information group for comorbidity totals. | |

| **Table S4.**  *Logistic Regression of Physical Health and Mental Health Comorbidities Predicting Likelihood of Reporting Loneliness at Baseline (n = 903)* | | | | |
| --- | --- | --- | --- | --- |
| **Predictor** | ***β*** | **OR** | **95% CI** | ***p*** |
| Mental Health Comorbidity | .66 | 1.95 | 1.48–2.56 | <.001 |
| Physical Health Comorbidity | .38 | 1.46 | 1.11–1.92 | .007 |
